# Laparoscopic vs open repair for primary midline ventral hernia: a prospective cohort study

**DOI:** 10.1007/s00423-023-02958-6

**Published:** 2023-08-08

**Authors:** Line Schjøth-Iversen, Mushegh A. Sahakyan, Xiaoran Lai, Arne Refsum

**Affiliations:** 1 Department of Surgery, Diakonhjemmet Hospital, Oslo, Norway; 2 The Intervention Center, Oslo University Hospital, Rikshospitalet, Oslo, Norway; 3 Department of Research & Development, Division of Emergencies and Critical Care, Oslo University Hospital, Oslo, Norway; 4 Department of Surgery N1, Yerevan State Medical University after M. Heratsi, Yerevan, Armenia; 5 Oslo Centre for Biostatistics and Epidemiology, University of Oslo, Oslo, Norway

**Keywords:** Hernia, Laparoscopy, Repair, IPOM, Recurrence

## Abstract

**Background:**

The optimal operative treatment for umbilical and epigastric hernia, i.e., primary midline ventral hernia (PMVH), is debatable. The most common techniques are the primary suture and open repair with mesh, while laparoscopic approach using intraperitoneally placed onlay mesh (IPOM) is less frequent. The aim of this study was to examine the outcomes of IPOM in PMVH. Perioperative results, recurrence, pain, and functional status were studied.

**Methods:**

This single-center prospective cohort study included consecutive patients with PMVH operated between September 2006 and December 2015. Systematic follow-up was conducted 6 months and 2 and 5 years postoperatively.

**Results:**

Seven hundred fifty-four patients underwent PMVH repair. Open repair without mesh, open repair with mesh, and IPOM were performed in 251 (34.9%), 273 (38%), and 195 (27.1%) patients, respectively. In the unmatched cohort, the incidence of postoperative complications was similar except postoperative seroma, which was more frequent after IPOM. The latter was also associated with longer length of stay. Open repair with mesh was associated with significantly lower recurrence compared with open repair without mesh and IPOM (5.2 vs 18.2 vs 13.8%, *p*=0.001, respectively). No differences were seen between the groups in terms of visual analog scale used for registering postoperative pain. These observations persisted after applying propensity score matching. In the multivariable analysis, open repair without mesh and IPOM significantly correlated with recurrence.

**Conclusions:**

In PMVH, open repair with mesh is associated with lower recurrence compared with open repair without mesh and IPOM. Pain, postoperative complications (except for seroma), and functional status are similar.

**Supplementary Information:**

The online version contains supplementary material available at 10.1007/s00423-023-02958-6.

## Introduction

The European Hernia Society classifies ventral hernias into primary and incisional [1]. Approximately 75% of ventral hernia repair in the USA are performed for primary ventral hernia, which comprises midline (epigastric and umbilical) and lateral (lumbar and spigelian) hernias [1, 2]. The surgical options in primary midline ventral hernia (PMVH) are primary suture or mesh repair. The use of mesh has been shown to reduce recurrence without increasing the incidence of surgical site infection (SSI), seroma, hematoma, and chronic pain [3–5], so it was recommended in the recently published guidelines [6].

The mesh can be placed either by open technique or laparoscopically by using intraperitoneal onlay mesh (IPOM). Cochrane database systematic review found that laparoscopic ventral repair was associated with a decreased risk of SSI and similar recurrence when compared with an open approach [7]. Liang and co-workers reported similar recurrence, fewer SSI, but more clinical cases of bulging [8]. At the same time, these studies have included a heterogenous group of patients with both primary and incisional hernia. In contrast, the role of IPOM in PMVH is less explored.

The aim of this study was to examine different repair techniques in patients with PMVH. The primary outcomes of interest were complications, recurrence, pain, and functional status.

## Methods

### Design and patient management

Single-center prospective cohort study was conducted. The local research ethics committee approved the study. All consecutive patients undergoing PMVH repair between September 2006 and December 2015 at our institution were suggested to participate in the study. Patients signed an informed consent and were subject to questionnaire-based follow-up 6 months, 2 years, and 5 years after surgery to capture an event of recurrence, level of pain, and change in the functional status. To capture an event of recurrence, all patients were asked about bulging or new hernia. Those with positive or indeterminate answers for recurrence or pain were summoned to our outpatient clinic for clinical examination or computed tomography (CT) examination. The exclusion criteria for this study included age under 18 years, presence of psychiatric disorders, not understanding Norwegian or English and rejection to participate (14 patients).

Patients were referred to our outpatient clinic and diagnosed by clinical examination and patient history. The indications for surgery were symptoms such as pain, discomfort, and impaired quality of life. Watchful waiting was applied if the patient had moderate symptoms or was at a significant risk for perioperative complications. There were no contraindications for surgery relative to smoking, body mass index (BMI), and other comorbidities. Preoperative radiological investigations were not performed routinely, but in cases where the diagnosis was uncertain, especially in adipose patients, or when several defects were suspected (Swiss cheese hernias) [2].

Outpatient surgery was performed, and the patients were discharged the same day unless they were classified as grades 3–4 according to the American Society of Anesthesiologists. Other possible reasons for longer hospital stay were living alone, living geographically far from the hospital, admission due to surgical complications, or expected postoperative pain.

### Surgical technique

The choice of the operative strategy was left to the surgeon performing or supervising the procedure. This was preoperatively discussed and agreed with the patient. Senior consultants performed most of the procedures. The rest were done by fellows with up to 3 years of surgical training supervised by attending surgeons.

A primary suture repair was performed using either resorbable or non-resorbable sutures. The defect was closed end-to-end or duplicated as in the Mayo technique. For an open repair with mesh, a mesh was placed either in the preperitoneal space or intraperitoneally. The type of mesh was either flat mesh or preformed patch. When the mesh was placed preperitoneally, the preperitoneal space was dissected with an overlap of 3 cm from the defect and the hernia sac mobilized. According to recent classification [9], the preperitonal plane is located between the transversalis fascia anteriorly and the peritoneum posteriorly. The mesh was sutured to the fascial defect. When placed intraperitoneally a patch was used, the hernia sac was opened and any adhesions released. The patch was placed underneath the peritoneum, the slips are fixed to the fascia. The closure of the fascial defect was at the discretion of the attending surgeon.

The laparoscopic repair was performed by using IPOM. Three ports were placed along the lateral aspect of the abdomen. Once hernia was identified and the content was reduced, the defect was closed at the discretion of the attending surgeon. The hernial sac was not excised. A coated mesh was then placed intraperitoneally with at least 3 cm of overlap from the margins of the defect. The mesh was secured by resorbable tacks with or without transfascial sutures.

Different meshes have been used during the study period. Due to the ongoing prospective registration, we noticed that Physiomesh (Ethicon US, LLC, Cincinnati, OH, USA) had higher recurrence and complication rates and hence was not used after 2015 (it was withdrawn from the market in May 2016).

### Definitions

PMVH is defined as a midline defect, i.e., either umbilical or epigastric hernia. The umbilical hernia is located with its center at the umbilicus, and the epigastric hernia is located with its center close to the midline above the umbilicus. Complications encompassed such postoperative events as SSI (superficial and deep infection), seroma, hematoma, ileus, bowel injury, and mortality. Long-term outcomes included hernia recurrence, pain, and functional status. Recurrence was defined as recurrence at the hernia site confirmed by clinical examination or radiological findings on a CT. Pain was recorded according to the visual analog scale (VAS), where the patients were asked to make a mark on a 10-cm straight line at a point that corresponds to the degree of pain. Accordingly, the VAS score was the distance in centimeters from the left end of the line to the patient’s mark. The value 0 represented no pain, while 10 represented worst possible pain. To measure functional status before and after surgery, a EuroQoL-5D questionnaire [10, 11] was used. The EQ-5D is a general health-related quality-of-life scale consisting of five questions concerning usual activity, self-care, mobility, pain or discomfort, and anxiety and depression. Each item has three possible levels (1, 2, or 3). The value 15 represented no impairment on daily life while 0 totally impairment on daily life.

### Statistics

Data were analyzed using R version 4.0.3. Categorical data were presented as percentages and were compared using chi-square test. Continuous data were presented as means and standard deviations (SDs) or medians and interquartile ranges (IQRs) as appropriate. An equality of mean/median test, analysis-of-variance (ANOVA) test, and Kruskal–Wallis test, respectively, was used to compare between-group differences. Pairwise comparisons between groups were corrected for multiple comparison by Holm’s method. All analyses were performed in both the total cohort and the propensity-score matched (PSM) cohort.

PSM (1:1:1) was applied for balancing the groups in terms of baseline characteristics and potentially minimizing confounding. Logistic regression was performed to estimate the propensity to undergo three different types of repair procedure regardless of the actual treatment received. Propensity scores were based on age, gender, body mass index (BMI), and hernia size and whether there are multiple hernia sites. Patients with any of these variables missing were excluded from the matching procedure. In order to perform matching with multiple treatments (*N*=3), vector matching on generalized propensity score was employed [12]. Caliper size of 0.25 multiplied by the standard deviation of generalized propensity score was specified. The R package “MatchIt” was used to create and assess the final matched cohort. Differences between the cohorts were assessed by using standardized mean difference.

## Results

**Fig. 1:**
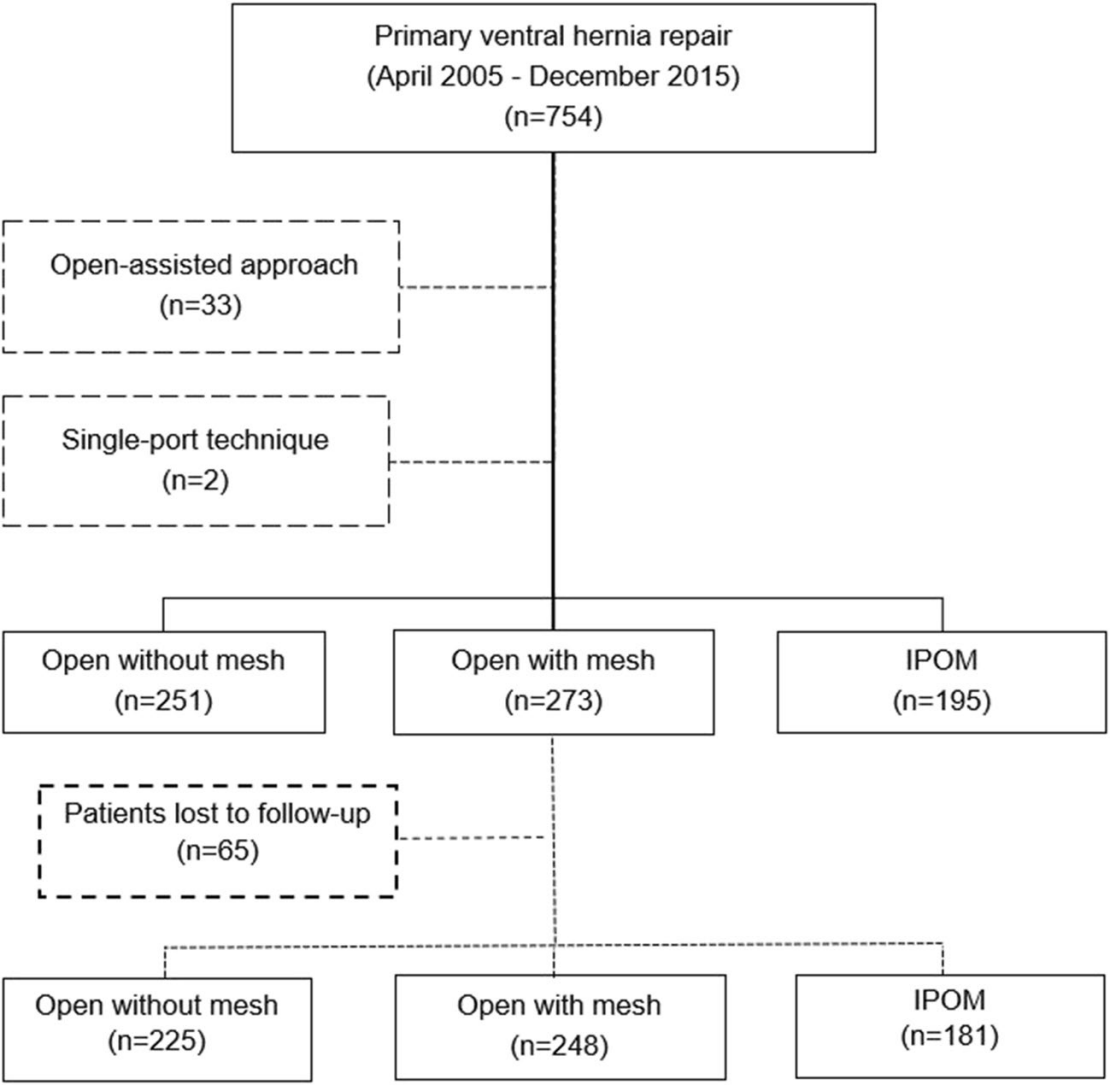
Study flow chart

A total number of 754 patients underwent repair of PMVH throughout the study period. Of these, 33 with open-assisted laparoscopic surgery and 2 with single-port repair were excluded (Fig. 1). Open repair without mesh, open repair with mesh, and IPOM were performed in 251 (34.9%), 273 (38%), and 195 (27.1%) patients, respectively. Patients lost to follow-up (*n*=65) were excluded from the analysis of long-term outcomes and PSM.

### Perioperative results

Open repair with mesh and IPOM were associated with older age and higher BMI compared with open repair without mesh (Table 1). The latter was more frequently applied in women. Hernia size greater than 4cm and the presence of multiple hernia significantly correlated with performing IPOM (11.9 vs 1.6 vs 0.4%, *p*=0.001 and 7.7 vs 3.3 vs 3.2%, *p*=0.039, respectively). It was associated with lower frequency of hernia defect closure (36.4 vs 87.2 vs 100%, *p*=0.001). After PSM, the differences in age, gender, BMI, and hernia characteristics became non-significant.

**Table 1:** Patient characteristics and intraoperative details for different surgical techniques used in primary ventral hernia repair

Parameters	Unmatched				PSM^0^ cohort			
	Open without mesh (*n*=251)	Open with mesh (*n*=273)	IPOM (*n*=195)	*p* value	Open without mesh (*n*=124)	Open with mesh (*n*=124)	IPOM (*n*=124)	*p* value
Age, years, mean (SD)	46.1 (14.9)	50.3 (13.3)	52.4 (14.8)	<0.01 *^, ┼^	49.6 (13.2)	48.2 (13.3)	48.1 (13.6)	0.55
Gender (female), *n* (%)	132 (52.6%)	87 (31.9%)	82 (42.1%)	<0.01*^, ┼, ╪^	63 (50.8%)	56 (45.2%)	64 (51.6%)	0.54
Body mass index, kg/m^2^, mean (SD)	23.9 (4.5)	29.2 (25.1)	29.6 (21.1)	<0.01 *^, ┼^	24.3 (3.4)	24.3 (2.8)	24.5 (4.1)	0.98
Hernia size > 4 cm, *n* (%)	1 (0.4%)	4 (1.5%)	20 (10.3%)	<0.01 ^┼, ╪^	0 (0%)	2 (1.6%)	1 (0.8%)	0.49
Multiple hernia, *n* (%)	8 (3.2%)	9 (3.3%)	15 (7.7%)	0.039 ^┼, ╪^	4 (3.2%)	6 (4.8%)	8 (6.5%)	0.5
Incarcerated hernia, *n* (%)	19 (7.6%)	12 (4.4%)	6 (3.1%)	0.08	5 (4%)	4 (3.2%)	4 (3.2%)	0.92
Preoperative function score, mean (SD)	12.6 (3.3)	13.3 (2.6)	12.7 (2.8)	0.02 ^¶,^ *	12.5 (3.4)	13.4 (2.3)	12.5 (3.1)	0.014 ^╪^
Mesh type, *n* (%)	-			<0.01				<0.01
Bard composix	-	170 (62.3%)	3 (1.5%)		-	94 (75.8%)	0 (0%)	
Proceed	-	3 (1.1%)	82 (42.3%)		-	0 (0%)	54 (43.5%)	
Ventralex	-	68 (24.9%)	0 (0%)		-	27 (21.8%)	0 (0%)	
Ventralight		0 (0%)	59 (30.4%)		-	0 (0%)	26 (21%)	
Other	-	32 (11.7%)	51 (25.8%)		-	3 (2.4%)	44 (35.5%)	
Mesh location, *n* (%)	-			<0.01				<0.01
Intraabdominal	-	149 (54.6%)	189 (96.9%)		-	67 (54%)	118 (95.2%)	
Preperitoneal	-	84 (30.8%)	2 (1%)		-	41 (33.1%)	0 (0%)	
Other	-	40 (14.7%)	4 (2.1%)		-	16 (12.9%)	6 (4.8%)	
Surgeon (trainee), *n* (%)	83 (33.1%)	85 (31.1%)	64 (32.8%)	0.88	39 (31.5%)	45 (36.3%)	42 (33.9%)	0.72
Defect closure, *n* (%)	251 (100%)	238 (87.2%)	71 (36.4%)	<0.01 ^┼, ╪^	124 (100%)	102 (82.3%)	42 (33.9%)	<0.01 *^, ┼, ╪^

*p < 0.05 between “open mesh +” and “open mesh—“; ^┼^ *p* < 0.05 between “IPOM” and “open mesh—“; ^╪^ *p* < 0.05 between “IPOM” and “open mesh + “; ^¶^Incomplete data; ^0^propensity score matched

The incidence of postoperative complications was similar except for postoperative seroma, which was more frequent after IPOM (Table 2). One case of postoperative mortality was observed after open repair without mesh. IPOM resulted in longer median length of stay compared with the other techniques (2 vs 1 vs 1, *p*<0.001). These differences persisted also after applying PSM.

**Table 2:** Postoperative results for different surgical techniques used in primary ventral hernia repair

Parameters	Unmatched				PSM^0^ cohort			
	Open without mesh (*n*=251)	Open with mesh (*n*=273)	IPOM (*n*=195)	*p* value	Open without mesh (*n*=124)	Open with mesh (*n*=124)	IPOM (*n*=124)	*p* value
Postoperative complications, *n* (%)	25 (10%)	40 (14.7%)	21 (10.8%)	0.21	12 (9.7%)	14 (11.3%)	13 (10.6%)	0.92
Superficial infection, *n* (%)	17 (6.8%)	12 (4.4%)	6 (3.1%)	0.18	10 (8.1%)	7 (5.6%)	3 (2.4%)	0.14
Deep infection, *n* (%)	1 (0.4%)	5 (1.8%)	2 (1%)	0.36	0 (0%)	1 (0.8%)	0 (0%)	0.37
Seroma, *n* (%)	0 (0%)	3 (1.1%)	6 (3.1%)	0.011^†^	0 (0%)	0 (0%)	4 (3.2%)	0.02 ^†, 0^
Hematoma, *n* (%)	2 (0.8%)	10 (3.7%)	2 (1%)	0.05	1 (0.8%)	3 (2.4%)	0 (0%)	0.17
Ileus, *n* (%)	0 (0%)	1 (0.4%)	1 (0.5%)	0.74	0 (0%)	1 (0.8%)	0 (0%)	0.37
Reoperation, *n* (%)	5 (2%)	6 (2.2%)	5 (2.6%)	0.95	1 (0.8%)	1 (0.8%)	0 (0%)	0.61
Mortality, *n* (%)	1 (0.4%)	0 (0%)	0 (0%)	0.62	0 (0%)	0 (0%)	0 (0%)	1.0
Postoperative stay, days, median (range)	1 (1–12)	1 (1-9)	2 (1–9)	<0.01^†, 0^	1 (1–6)	1 (1–4)	2 (1–3)	<0.01 ^†, 0^
Postoperative pain score, mean (SD) ^¶^	1.1 (1.7)	1.4 (1.8)	1.5 (1.9)	0.22	1.1 (1.6)	1.7 (2.2)	1.3 (1.6)	0.32

*p < 0.05 between “open mesh +” and “open mesh—“; ^†^*p* < 0.05 between “laparoscopic” and “open mesh—“; ^0^*p* < 0.05 between “laparoscopic” and “open mesh + “;^¶^Incomplete data; ^0^Propensity score matched

### Long-term results

Hernia recurrence was observed in 79 (12.1%) cases. Its incidence did not significantly change throughout the study period both in the total cohort and in the 3 groups. Recurrence rates, as well as pain and functional score at different time points following surgery are presented in Table 3. In the unmatched cohort, open repair with mesh was associated with significantly lower recurrence compared with open repair without mesh and IPOM (5.2 vs 18.2 vs 13.8%, *p*=0.001, respectively). While no statistically significant differences were found between the groups 6 months after surgery, open repair without mesh resulted in higher recurrence at 2- and 5-year follow-up. No differences were seen between the groups in terms of VAS pain score. Significant difference in functional score was observed between open repair with mesh and IPOM 2 years after surgery; however, it was not present at 5-year follow-up.

**Table 3:** Results of the long-term follow-up after primary ventral hernia repair by using different surgical techniques

Parameters	Unmatched				PSM^0^ cohort			
	Open without mesh (*n*=225)	Open with mesh (*n*=248)	IPOM (*n*=181)	*p* value	Open without mesh (*n*=124)	Open with mesh (*n*=124)	IPOM (*n*=124)	*p* value
Recurrence, *n* (%)	41 (18.2%)	13 (5.2%)	25 (13.8%)	<0.01*^,^ †	18 (14.5%)	5 (4%)	14 (11.3%)	0.02 *
Recurrence at 6 months, *n*	15	6	8	0.2	8	1	9	0.03 *, †
Recurrence at 2 years, *n*	16	5	10	0.04 *	9	4	3	0.11
Recurrence at 5 years, *n*	10	2	7	0.01 *	1	0	2	0.32
Pain score (6 months), mean (SD) ^¶^	0.48 (1.08)	0.7 (1.43)	0.78 (1.7)	0.2	0.52 (1.19)	0.73 (1.58)	0.73 (1.47)	0.55
Pain score (2 years), mean (SD) ^¶^	0.49 (1.1)	0.49 (1.1)	0.59 (1.2)	0.69	0.55 (1.22)	0.62 (1.22)	0.42 (0.99)	0.07
Pain score (5 years), mean (SD) ^¶^	0.37 (0.96)	0.39 (0.96)	0.54 (1.3)	0.33	0.31 (0.9)	0.35 (0.63)	0.21 (0.6)	0.1
Functional score (6 months), mean (SD) ^¶^	14.5 (1.8)	14.4 (1.9)	14.4 (1.6)	0.79	14.7 (0.9)	14.7 (0.8)	14.5 (0.97)	0.25
Functional score (2 years), mean (SD) ^¶^	14.4 (2.1)	14.7 (0.8)	14.1 (2.5)	0.02 ^†^	14.6 (0.91)	14.7 (0.76)	14.8 (0.59)	0.08
Functional score (5 years), mean (SD) ^¶^	14.5 (1.9)	14.6 (1.6)	14.3 (1.7)	0.44	14.8 (0.55)	14.9 (0.52)	14.8 (0.62)	0.64

*p < 0.05 between “open mesh +” and “open mesh—“; †*p* < 0.05 between “laparoscopic” and “open mesh + “; ^¶^Incomplete data; ^0^Propensity score matched

After PSM the incidence of hernia recurrence remained significantly different between the open repair with and without mesh (4 vs 14.5%, *p*=0.01, respectively). Six months after surgery, the recurrence rate was significantly lower for open repair with mesh compared with open repair without mesh and IPOM. Similar results were observed at 2- and 5-year follow-up. VAS and functional score were comparable between the groups.

Preoperative function score, trainee as an operator and repair method were associated with PMVH recurrence in the univariable model (Table 4). The multivariable analyses demonstrated that low preoperative function score, as well as IPOM and open repair without mesh were associated with recurrence.

**Table 4:** Uni- and multivariable analysis of risk factors for recurrence following primary ventral hernia repair

Parameters	Univariable analysis			Multivariable analysis	
	Recurrence (*n*=79)	No recurrence (*n*=575)	*p* value	Odds ratio (95% CI)	*p* value
Age, years, mean (SD)	46.9 (13.4)	50.3 (14.4)	0.35		
Gender (female), *n* (%)	34 (43%)	239 (41.6%)	0.8		
Body mass index, kg/m^2^, mean (SD)	26.1 (4.9)	27.9 (21.6)	0.32		
Hernia size > 4 cm, *n* (%)	5 (6.3%)	17 (2.9%)	0.17		
Multiple hernia, *n* (%)	75 (94.9%)	549 (95.6%)	0.77		
Incarcerated hernia, *n* (%)	4 (5.1%)	28 (4.9%)	1.0		
Preoperative function score, mean (SD)	11.5 (4.2)	13.1 (2.6)	< 0.01	0.88 (0.81–0.94)	< 0.01
Surgeon (trainee), *n* (%)	33 (41.8%)	178 (31%)	0.05	1.5 (0.86–2.61)	0.15
Defect closure, *n* (%)	17 (21.5%)	130 (22.6%)	0.83		
Repair technique, *n* (%)			< 0.01		
Open mesh+	13 (16.5%)	235 (40.9%)		baseline	
Open mesh-	41 (51.9%)	184 (32%)		4.11 (1.94–8.72)	0.001
IPOM*	25 (31.6%)	156 (27.1%)		3.36 (1.55–7.27)	0.002
Postoperative complications, *n* (%)	10 (12.7%)	65 (11.3%)	0.72		
Surgical site infection, *n* (%)	3 (3.8%)	34 (5.9%)	0.61		
Reoperation, *n* (%)	3 (3.8%)	13 (2.3%)	0.43		

*Intraperitoneal onlay mesh

### Subgroup analyses

Subgroup analyses focused on elderly (≥ 70 years old) patients, those not receiving Physiomesh (Ethicon US, LLC, Cincinnati, OH, USA), as well as on performances of the consultant surgeons.

Preoperative data were comparable in the elderly patients (suppl. table 1a). Hernia defect closure was least frequent in IPOM compared with open repair with and without mesh. No statistically significant differences were observed between the groups in terms of postoperative and long-term outcomes (suppl. table 1b).

After having excluded the cases where the Physiomesh (Ethicon US, LLC, Cincinnati, OH, USA) was used, perioperative outcomes from the unmatched analysis of the total cohort did not change (suppl. table 2a). Analysis of long-term outcomes demonstrated that open repair with mesh was associated with lower incidence of hernia recurrence than open repair without mesh at any time point (suppl. table 2b). As for the IPOM, it did not result in significantly higher incidence of recurrence compared with open repair with mesh.

Finally, perioperative results of the procedures performed exclusively by senior consultants did not significantly differ from those observed in the total cohort (suppl. table 3). Total recurrence rate was significantly lower after open repair with mesh compared to IPOM and open repair without mesh. At 5-year follow-up, the incidence of hernia recurrence was higher after open repair without mesh compared to the repair with mesh.

## Discussion

In the current study, the recurrence rate after IPOM was higher than after open repair with mesh, but lower compared with open repair without mesh. Furthermore, open repair without mesh, IPOM, and low preoperative function score were associated with recurrence following surgery for PMVH. Interestingly, these findings do not correlate with the literature data. A meta-analysis from Hajibandeh and co-workers found that laparoscopic repair for umbilical and paraumbilical hernia was associated with a lower risk of wound infection and recurrence, but the overall evidence level was low [13]. Others have reported similar recurrence rates for open and laparoscopic repair [2].

There can be several reasons behind our findings. First, different meshes have been used during the study period. Physiomesh (Ethicon US, LLC, Cincinnati, OH, USA) was not used after 2015 due to high recurrence observed by our internal audit. That was in line with the literature reporting increased rate of complications and recurrence with the use of Physiomesh (Ethicon US, LLC, Cincinnati, OH, USA) [14]. Therefore, we performed subgroup analysis after having excluding cases with Physiomesh (Ethicon US, LLC, Cincinnati, OH, USA). However, it did not significantly affect higher recurrence rate following IPOM compared with open repair with mesh. Another possible reason for high recurrence after IPOM can be technical challenges associated with mesh fixation on the trocar side (narrow space, lack of angulation of the fixating device). As a result, the mesh can be displaced from its correct site. Finally, in a recently published randomized trial, closure of the fascial defect in umbilical hernia IPOM repair was shown to significantly reduce long-term recurrence [15]. In the current study, closure of the fascial defect in the IPOM group was performed only in 36.4% of cases.

Although about one-third of the procedures reported in this study were performed by fellow surgeons with a few years of surgical training, surgeon experience per se was not associated with recurrence. This finding may be a result of focus on team driven surgery with sufficient training and a low threshold to ask for assistance or an intraoperative second opinion even among the most experienced surgeons. We believe experienced assistance is crucial levelling out the outcome differences from the inexperienced surgeons. Køckerling et al. emphasizes the importance of tailored approach in hernia surgery where several techniques must be taught to promote a better outcome with minimal morbidity to each patient [16].

The incidence of postoperative complications was similar except seroma which was more frequent after IPOM. This is in line with the findings from Christoffersen et al. who reported significantly lower seroma formation rate in the defect closure group compared with the no closure [15]. Notably, one of the most feared complications of IPOM, namely ileus, was observed in only one patient (0.5%). The last years there has subsequently been an increasing focus on patient-centered outcomes and quality of life in hernia treatment [17, 18]. Veenendaal et al. reported a high percentage (53%) of persistent symptoms or pain 3 years after incisional hernia repair [19]. In the current study, the functional score at 6 months, 2 years, and 5 years postoperatively are significantly higher than the preoperative score in all 3 groups. Asencio et al. reported no differences in pain or EQ5D QoL after laparoscopic versus open incisional hernia repair; however, the follow-up was short (1 year) [11]. In the current study, a significant difference in functional score was observed between open repair with mesh and IPOM 2 years after surgery, but the difference became non-significant after applying PSM.

Fixating the mesh to the abdominal wall with tacks and/or transfascial sutures has been linked to increased postoperative pain [2, 7]. However, studies found no difference in pain between open and laparoscopic repair [11, 20]. This is in line with our findings. Køckerling et al. reported high incidence of postoperative pain following IPOM for epigastric hernia repair at 1-year follow-up [21]. However, in the current study, pain score was similarly low in the three groups throughout the first 5 years if surgery.

To the best of our knowledge, this is the first prospective study addressing different repair techniques for PMVH and including over 700 patients with a 5-year follow-up period. As of now, the literature on umbilical and epigastric hernias is limited in both quantity and quality. Furthermore, most systematic reviews have used pooled data from incisional and primary hernias in their analysis which according to Stabilini et al. should no longer be acceptable since primary ventral hernias and incisional hernias are different conditions [22]. Subramanian recommends separating primary hernias from secondary hernias when evaluating surgical outcomes [23].

In the latest guidelines, it was recommended to place the mesh preperitonealy during ventral hernia repair to avoid bowel injury and mesh-related complications, as well as to reduce the risk of recurrence (6). This can also be achieved with newly emerging minimally invasive techniques such as robotic surgery, e-TEP (extended totally extraperitoneal approach), e-MILOS (endoscopic minimal less open sublay repair), and totally endoscopic sublay repair [24, 25]. However, there is still insufficient data to suggest the superiority of one technique over another (6). At the same time, Køckerling et al. showed that the proportion of IPOM in epigastric hernia repair declined between 2013 and 2019 from 26.0 to 18.2%, while open mesh repair and the new innovative techniques increased [21].

The current study had several limitations. First, the size of the defect was classified as larger or smaller than 4 cm and with several defects or not. Even if the size of the mesh was prospectively registered, the lack of more detailed hernia defect size precludes mesh-to-hernia size ratio analysis. Second, surgical techniques were not standardized (closure or not of the defect, the use of different meshes, placement of the mesh). Third, 65 (9%) patients were lost to follow-up, so their long-term results remain unknown (also 14 patients rejected to sign the informed consent). Finally, the use of patient-based questionnaires to capture an event of recurrence remains uncertain and debated. Physical examination was performed in cases when recurrence was suspected or remained uncertain upon receipt of the completed questionnaires.

Our findings indicate that the recurrence rate after PMVH is lowest following open repair with mesh, while the use of IPOM was associated with recurrence. VAS, complications (except for seroma), and functional status were similar. Further data is needed to understand if the disadvantages of IPOM can be overcome by the emerging new minimally-invasive techniques.

### Supplementary information

ESM 1 (DOCX 17 kb)

ESM 2 (DOCX 18 kb)

ESM 3 (DOCX 17 kb)

## References

[1] Muysoms FE, Miserez M, Berrevoet F, Campanelli G, Champault GG, Chelala E, Dietz UA, Eker HH, El Nakadi I, Hauters P, Hidalgo Pascual M, Hoeferlin A, Klinge U, Montgomery A, Simmermacher RK, Simons MP, Smietański M, Sommeling C, Tollens T, Vierendeels T, Kingsnorth A (2009). Classification of primary and incisional abdominal wall hernias. Hernia:J Hernias Abdom. Wall Surg.

[2] Van Hoef S, Tollens T (2019). Primary non-complicated midline ventral hernia: is laparoscopic IPOM still a reasonable approach?. Hernia.

[3] Kaufmann R, Halm JA, Eker HH, Klitsie PJ, Nieuwenhuizen J, van Geldere D, Simons MP, van der Harst E, van 't Riet M, van der Holt B, Kleinrensink GJ, Jeekel J, Lange JF (2018) Mesh versus suture repair of umbilical hernia in adults: a randomised, double-blind, controlled, multicentre trial. Lancet (London, England) 391**:**860-869 10.1016/S0140-6736(18)30298-8 29459021

[4] Alkhatib H, Fafaj A, Olson M, Stewart T, Krpata DM (2019). Primary uncomplicated midline ventral hernias: factors that influence and guide the surgical approach. Hernia.

[5] Bisgaard T, Kaufmann R, Christoffersen MW, Strandfelt P, Gluud LL (2019). Lower risk of recurrence after mesh repair versus non-mesh sutured repair in open umbilical hernia repair: a systematic review and meta-analysis of randomized controlled trials. Scand J Surg: SJS: Off Organ Finn Surg Soc Scand Surg Soc.

[6] Henriksen NA, Montgomery A, Kaufmann R, Berrevoet F, East B, Fischer J, Hope W, Klassen D, Lorenz R, Renard Y, Garcia Urena MA, Simons MP (2020). Guidelines for treatment of umbilical and epigastric hernias from the European Hernia Society and Americas Hernia Society. Br J Surg.

[7] Sauerland S, Walgenbach M, Habermalz B, Seiler CM, Miserez M (2011) Laparoscopic versus open surgical techniques for ventral or incisional hernia repair. Cochrane Database Syst Rev (3):CD007781. 10.1002/14651858.CD007781 10.1002/14651858.CD007781.pub2 PMC13389912 21412910

[8] Liang MK, Berger RL, Li LT, Davila JA, Hicks SC, Kao LS (2013). Outcomes of laparoscopic vs open repair of primary ventral hernias. JAMA Surg.

[9] Parker SG, Halligan S, Liang MK, Muysoms FE, Adrales GL, Boutall A, de Beaux AC, Dietz UA, Divino CM, Hawn MT, Heniford TB, Hong JP, Ibrahim N, Itani KMF, Jorgensen LN, Montgomery A, Morales-Conde S, Renard Y, Sanders DL, Smart NJ, Torkington JJ, Windsor ACJ (2020). International classification of abdominal wall planes (ICAP) to describe mesh insertion for ventral hernia repair. Br J Surg.

[10] EuroQol Group (1990) EuroQol--a new facility for the measurement of health-related quality of life. Health Policy 16(3):199–208 10.1016/0168-8510(90)90421-9 10109801

[11] Asencio F, Aguiló J, Peiró S, Carbó J, Ferri R, Caro F, Ahmad M (2009). Open randomized clinical trial of laparoscopic versus open incisional hernia repair. Surg Endosc.

[12] Lopez MJ, Gutman R (2017) Estimation of causal effects with multiple treatments: a review and new ideas. Stat Sci pp 432–454

[13] Hajibandeh S, Hajibandeh S, Sreh A, Khan A, Subar D, Jones L (2017). Laparoscopic versus open umbilical or paraumbilical hernia repair: a systematic review and meta-analysis. Hernia.

[14] Helgstrand F, Thygesen LC, Bisgaard T, Jørgensen LN, Friis-Andersen H (2020). Differential recurrence after laparoscopic incisional hernia repair: importance of a nationwide registry-based mesh surveillance. Br J Surg.

[15] Christoffersen MW, Westen M, Rosenberg J, Helgstrand F, Bisgaard T (2020). Closure of the fascial defect during laparoscopic umbilical hernia repair: a randomized clinical trial. Br J Surg.

[16] Köckerling F, Sheen AJ, Berrevoet F, Campanelli G, Cuccurullo D, Fortelny R, Friis-Andersen H, Gillion JF, Gorjanc J, Kopelman D, Lopez-Cano M, Morales-Conde S, Österberg J, Reinpold W, Simmermacher RKJ, Smietanski M, Weyhe D, Simons MP (2019). The reality of general surgery training and increased complexity of abdominal wall hernia surgery. Hernia.

[17] Langbach O, Bukholm I, Benth J, Røkke O (2016). Long-term quality of life and functionality after ventral hernia mesh repair. Surg Endosc.

[18] Colavita PD, Tsirline VB, Belyansky I, Walters AL, Lincourt AE, Sing RF, Heniford BT (2012). Prospective, long-term comparison of quality of life in laparoscopic versus open ventral hernia repair. Ann Surg.

[19] van Veenendaal N, Poelman MM, van den Heuvel B, Dwars BJ, Schreurs WH, Stoot J, Bonjer HJ (2021). Patient-reported outcomes after incisional hernia repair. Hernia.

[20] Wassenaar E, Schoenmaeckers E, Raymakers J, van der Palen J, Rakic S (2010). Mesh-fixation method and pain and quality of life after laparoscopic ventral or incisional hernia repair: a randomized trial of three fixation techniques. Surg Endosc.

[21] Köckerling F, Adolf D, Zarras K, Fortelny R, Lorenz R, Lammers B, Reinpold W, Stechemesser B, Schug-Pass C, Weyhe D (2021). What is the reality in epigastric hernia repair?-a trend analysis from the Herniamed Registry. Hernia.

[22] Stabilini C, Cavallaro G, Dolce P, Capoccia Giovannini S, Corcione F, Frascio M, Sodo M, Merola G, Bracale U (2019). Pooled data analysis of primary ventral (PVH) and incisional hernia (IH) repair is no more acceptable: results of a systematic review and metanalysis of current literature. Hernia.

[23] Subramanian A, Clapp ML, Hicks SC, Awad SS, Liang MK (2013). Laparoscopic ventral hernia repair: primary versus secondary hernias. J Surg Res.

[24] Reinpold W, Schröder M, Berger C, Nehls J, Schröder A, Hukauf M, Köckerling F, Bittner R (2019). Mini- or Less-open Sublay Operation (MILOS): a new minimally invasive technique for the extraperitoneal mesh repair of incisional hernias. Ann Surg.

[25] Reinpold W, Schröder M, Berger C, Stoltenberg W, Köckerling F (2019). MILOS and EMILOS repair of primary umbilical and epigastric hernias. Hernia.

