# Supplementary material for: Laparoscopic vs open repair for primary midline ventral hernia: a prospective cohort study
Source: Langenbecks Arch Surg. 2023 Aug 8;408(1):300. doi: 10.1007/s00423-023-02958-6 (PMC10409826; doi:10.1007/s00423-023-02958-6)
Supplement: Supplementary file 1 — (DOCX 17 kb) [file 423_2023_2958_MOESM1_ESM.docx]

**Supplementary table 1. Subgroup analysis in patients aged ≥ 70 years undergoing primary midline ventral hernia repair by different surgical techniques: a) short-term outcomes, b) long-term outcomes.**

a)

| **Parameters** | **Open without mesh**  **(n=20)** | **Open with mesh**  **(n=22)** | **IPOM**  **(n=23)** | **p-value** |
| --- | --- | --- | --- | --- |
| Gender (female) | 5 | 2 | 7 | 0.2 |
| Body mass index, kg/m^2^, mean (SD) | 25.9 (4.3) | 26.1 (5.2) | 26.5 (6.3) | 0.94 |
| Hernia size > 4 cm | 0 | 1 | 2 | 0.77 |
| Multiple hernia | - | - | - | **-** |
| Incarcerated hernia | 3 | 4 | 1 | 0.37 |
| Preoperative VAS score, mean (SD) ^¶^ | 12.4 (3.4) | 11.9 (3.9) | 12.7 (3.3) | 0.8 |
| Surgeon (trainee) | 3 | 3 | 9 | 0.09 |
| Defect closure | 20 | 17 | 10 | <0.01^┼, ╪^ |
| Postoperative complications | 4 | 4 | 2 | 0.58 |
| Superficial infection | 1 | 0 | 1 | 0.76 |
| Deep infection | 1 | 0 | 0 | 0.31 |
| Seroma | - | - | - | **-** |
| Hematoma | 1 | 2 | 0 | 0.4 |
| Ileus | 0 | 0 | 1 | 1.0 |
| Reoperation | 1 | 0 | 2 | 0.42 |
| Mortality | - | - | - | - |
| Postoperative stay, days, median (range) | 2 (1-12) | 2 (1-9) | 2 (1.6) | 0.09 |
| VAS pain score, mean (SD) ^¶^ | 0.46 (0.88) | 1.1 (1.4) | 1.2 (1.3) | 0.28 |

^┼^ p < 0.05 between “IPOM” and “open mesh – “; ^╪^ p < 0.05 between “IPOM” and “open mesh + “

b)

| **Parameters** | **Open without mesh**  **(n=20)** | **Open with mesh**  **(n=19)** | **IPOM**  **(n=18)** | **p-value** |
| --- | --- | --- | --- | --- |
| Recurrence | 1 | 0 | 1 | 0.76 |
| Recurrence at 6 months | 1 | 0 | 0 | 1.0 |
| Recurrence at 2 years | 0 | 0 | 1 | 0.31 |
| Recurrence at 5 years | 1 | 0 | 0 | 0.63 |
| VAS pain score (6 months), mean (SD) ^¶^ | 0.65 (1.93) | 0.29 (0.61) | 1 (1.97) | 0.51 |
| VAS pain score (2 years), mean (SD) ^¶^ | 0.07 (0.26) | 0.36 (1.08) | 0.59 (0.88) | 0.25 |
| VAS pain score (5 years), mean (SD) ^¶^ | 0.62 (1.94) | 0.36 (0.93) | 0.5 (0.91) | 0.88 |
| VAS functional score (6 months), mean (SD) ^¶^ | 13.6 (3.6) | 14.6 (0.8) | 14.3 (1.4) | 0.54 |
| VAS functional score (2 years), mean (SD) ^¶^ | 12.2 (5.4) | 14.7 (0.5) | 13.5 (3.5) | 0.19 |
| VAS functional score (5 years), mean (SD) ^¶^ | 13.1 (3.9) | 14.6 (0.8) | 14.3 (1.2) | 0.27 |
