# Supplementary material for: Laparoscopic vs open repair for primary midline ventral hernia: a prospective cohort study
Source: Langenbecks Arch Surg. 2023 Aug 8;408(1):300. doi: 10.1007/s00423-023-02958-6 (PMC10409826; doi:10.1007/s00423-023-02958-6)
Supplement: Supplementary file 2 — (DOCX 18 kb) [file 423_2023_2958_MOESM2_ESM.docx]

**Supplementary table 2. Subgroup analysis among the patients who have not received Physiomesh during primary midline ventral hernia repair by different surgical techniques: a) short-term outcomes, b) long-term outcomes.**

a)

| **Parameters** | **Open without mesh**  **(n=251)** | **Open with mesh**  **(n=260)** | **IPOM**  **(n=155)** | **p-value** |
| --- | --- | --- | --- | --- |
| Age, years, mean (SD) | 46.1 (14.9) | 50.3 (13.4) | 52.8 (15.1) | <0.01*^, ┼^ |
| Gender (female), n (%) | 132 (52.6%) | 85 (32.7%) | 66 (42.6%) | <0.01*^, ┼, ╪^ |
| Body mass index, kg/m^2^, mean (SD) | 23.9 (4.5) | 29.4 (25.3) | 30.2 (23.6) | <0.01*^, ┼^ |
| Hernia size > 4 cm, n (%) | 1 (0.4%) | 4 (1.7%) | 18 (13.8%) | <0.01^┼, ╪^ |
| Multiple hernia, n (%) | 8 (3.2%) | 7 (2.7%) | 13 (8.4%) | 0.022^┼, ╪^ |
| Incarcerated hernia, n (%) | 19 (7.6%) | 11 (4.2%) | 6 (3.9%) | 0.16 |
| Preoperative VAS score, mean (SD)^¶^ | 12.6 (3.3) | 13.3 (2.6) | 12.6 (2.7) | 0.016 * |
| Surgeon (trainee), n (%) | 83 (33.1%) | 82 (31.5%) | 47 (30.3%) | 0.84 |
| Defect closure, n (%) | 251 (100%) | 237 (91.2%) | 58 (37.4%) | <0.01^┼, ╪^ |
| Postoperative complications, n (%) | 25 (10%) | 36 (13.8%) | 17 (11%) | 0.37 |
| Superficial infection, n (%) | 17 (6.8%) | 12 (4.6%) | 5 (3.2%) | 0.26 |
| Deep infection, n (%) | 1 (0.4%) | 5 (1.9%) | 2 (1.3%) | 0.35 |
| Seroma, n (%) | 0 (0%) | 0 (0%) | 4 (2.6%) | <0.01^┼, ╪^ |
| Hematoma, n (%) | 2 (0.8%) | 9 (3.5%) | 2 (1.3%) | 0.1 |
| Ileus, n (%) | 0 (0%) | 1 (0.4%) | 0 (0%) | 1.0 |
| Reoperation, n (%) | 5 (2%) | 5 (1.9%) | 4 (2.6%) | 0.5 |
| Mortality, n (%) | 1 (0.4%) | 0 (0%) | 0 (0%) | 0.61 |
| Postoperative stay, days, median (range) | 1 (1-12) | 1 (1-9) | 2 (1-9) | <0.01^┼, ╪^ |
| VAS pain score, mean (SD) ^¶^ | 1.1 (1.7) | 1.4 (1.9) | 1.6 (1.9) | 0.14 |

* p < 0.05 between “open mesh +” and “open mesh – “; ^┼^ p < 0.05 between “IPOM” and “open mesh – “;

^╪^ p < 0.05 between “IPOM” and “open mesh + “; ^¶^ incomplete data

b)

| **Parameters** | **Open without mesh**  **(n=225)** | **Open with mesh**  **(n=237)** | **IPOM**  **(n=144)** | **p-value** |
| --- | --- | --- | --- | --- |
| Recurrence, n (%) | 41 (18.2%) | 13 (5.5%) | 15 (10.4%) | <0.01 *^,^ ^╪^ |
| Recurrence at 6 months, n (%) | 15 (6.7%) | 6 (2.6%) | 3 (2.1%) | 0.03 *^, ╪^ |
| Recurrence at 2 years, n (%) | 16 (7.1%) | 5 (2.1%) | 8 (5.6%) | 0.03 * |
| Recurrence at 5 years, n (%) | 11 (4.9%) | 2 (0.8%) | 4 (2.8%) | 0.011* |
| VAS pain score (6 months), mean (SD) ^¶^ | 0.48 (1.08) | 0.71 (1.44) | 0.8 (1.74) | 0.19 |
| VAS pain score (2 years), mean (SD) ^¶^ | 0.49 (1.09) | 0.49 (1.11) | 0.6 (1.24) | 0.69 |
| VAS pain score (5 years), mean (SD) ^¶^ | 0.37 (0.96) | 0.4 (0.97) | 0.57 (1.36) | 0.27 |
| VAS functional score (6 months), mean (SD) ^¶^ | 14.5 (1.8) | 14.4 (1.9) | 14.4 (1.7) | 0.77 |
| VAS functional score (2 years), mean (SD) ^¶^ | 14.4 (2.1) | 14.7 (0.8) | 14.1 (2.5) | 0.021† |
| VAS functional score (5 years), mean (SD) ^¶^ | 14.5 (1.9) | 14.6 (1.6) | 14.2 (1.9) | 0.19 |

* p < 0.05 between “open mesh +” and “open mesh – “; † p < 0.05 between “IPOM” and “open mesh + “;

^╪^ p < 0.05 between “IPOM” and “open mesh - “; ^¶^ incomplete data.
