# Supplementary material for: Laparoscopic vs open repair for primary midline ventral hernia: a prospective cohort study
Source: Langenbecks Arch Surg. 2023 Aug 8;408(1):300. doi: 10.1007/s00423-023-02958-6 (PMC10409826; doi:10.1007/s00423-023-02958-6)
Supplement: Supplementary file 3 — (DOCX 17 kb) [file 423_2023_2958_MOESM3_ESM.docx]

**Supplementary table 3. Subgroup analysis among consultant surgeons performing primary midline ventral hernia repair by using different surgical techniques: a) short-term outcomes, b) long-term outcomes.**

a)

| **Parameters** | **Open without mesh**  **(n=168)** | **Open with mesh**  **(n=188)** | **IPOM**  **(n=131)** | **p-value** |
| --- | --- | --- | --- | --- |
| Age, year, mean (SD) | 46.7 (15.8) | 51.4 (13.6) | 52.5 (14.6) | 0.01*^, ┼^ |
| Gender (female), n (%) | 92 (54.8%) | 55 (29.3%) | 57 (43.5%) | <0.01*^, ┼, ╪^ |
| Body mass index, kg/m^2^, mean (SD) | 23.6 (4.5) | 30.3 (29.9) | 28.5 (15.4) | 0.014*^, ┼^ |
| Hernia size > 4 cm, n (%) | 0 (0%) | 2 (1.2%) | 15 (13.4%) | <0.01^┼, ╪^ |
| Multiple hernia, n (%) | 6 (3.6%) | 5 (2.7%) | 10 (7.6%) | 0.08 |
| Incarcerated hernia, n (%) | 14 (8.3%) | 8 (4.3%) | 4 (3.1%) | 0.09 |
| Preoperative VAS score, mean (SD) ^¶^ | 12.4 (3.4) | 13.3 (2.6) | 12.7 (2.8) | 0.043* |
| Defect closure ^┼, ╪^ | 168 (100%) | 160 (85.1%) | 51 (38.9%) | <0.01^┼, ╪^ |
| Postoperative complications, n (%) | 19 (11.3%) | 26 (13.8%) | 17 (13%) | 0.77 |
| Superficial infection, n (%) | 12 (7.1%) | 9 (4.8%) | 5 (3.8%) | 0.41 |
| Deep infection, n (%) | 1 (0.6%) | 3 (1.6%) | 2 (1.5%) | 0.67 |
| Seroma, n (%) | 0 (0%) | 3 (1.6%) | 6 (4.6%) | 0.01 |
| Hematoma, n (%) | 2 (1.2%) | 6 (3.2%) | 2 (1.5%) | 0.41 |
| Ileus, n (%) | 0 (0%) | 1 (0.5%) | 0 (0%) | 1.0 |
| Reoperation, n (%) | 3 (1.8%) | 4 (2.1%) | 4 (3.1%) | 0.85 |
| Mortality, n (%) | 1 (0.6%) | 0 (0%) | 0 (0%) | 0.61 |
| Postoperative stay, days, median (range) | 1 (1-12) | 1 (1-9) | 2 (1-9) | < 0.001 |
| VAS pain score, mean (SD) ^¶^ | 1.1 (1.8) | 1.3 (1.8) | 1.6 (2.1) | 0.42 |

* p < 0.05 between “open mesh +” and “open mesh – “; † p < 0.05 between “IPOM” and “open mesh + “;

^╪^ p < 0.05 between “IPOM” and “open mesh - “; ^¶^ incomplete data.

b)

| **Parameters** | **Open without mesh**  **(n=148)** | **Open with mesh**  **(n=172)** | **IPOM**  **(n=123)** | **p-value** |
| --- | --- | --- | --- | --- |
| Recurrence, n (%) | 22 (14.9%) | 9 (5.2%) | 15 (12.2%) | 0.014*^, ┼^ |
| Recurrence at 6 months, n (%) | 9 | 5 | 5 | 0.13 |
| Recurrence at 2 years, n (%) | 7 | 3 | 7 | 0.35 |
| Recurrence at 5 years, n (%) | 7 | 1 | 3 | 0.08* |
| VAS pain score (6 months), mean (SD) ^¶^ | 0.48 (1.1) | 0.77 (1.6) | 0.82 (1.75) | 0.24 |
| VAS pain score (2 years), mean (SD) ^¶^ | 0.55 (1.15) | 0.53 (1.24) | 0.58 (1.29) | 0.96 |
| VAS pain score (5 years), mean (SD) ^¶^ | 0.42 (1.1) | 0.41 (1.03) | 0.48 (1.08) | 0.89 |
| VAS functional score (6 months), mean (SD) ^¶^ | 14.4 (2.1) | 14.2 (2.3) | 14.2 (1.9) | 0.68 |
| VAS functional score (2 years), mean (SD) ^¶^ | 14.2 (2.4) | 14.7 (0.8) | 13.9 (2.7) | 0.044^┼^ |
| VAS functional score (5 years), mean (SD) ^¶^ | 14.4 (2.1) | 14.5 (1.9) | 14.2 (1.9) | 0.66 |

* p < 0.05 between “open mesh +” and “open mesh – “; † p < 0.05 between “IPOM” and “open mesh + “; ^¶^ incomplete data.
